# Co-expression of long non-coding RNAs and autism risk genes in the developing human brain

**DOI:** 10.1186/s12918-018-0639-x

**Published:** 2018-12-14

**Authors:** Steven B. Cogill, Anand K. Srivastava, Mary Qu Yang, Liangjiang Wang

**Affiliations:** 1 Department of Genetics and Biochemistry, Clemson University, Clemson, SC 29646 USA; 2 J.C. Self Research Institute of Human Genetics, Greenwood Genetic Center, Greenwood, SC 29646 USA; 3 MidSouth Bioinformatics Center, Department of Information Science, University of Arkansas at Little Rock, Little Rock, AR 72204 USA

**Keywords:** Autism, lncRNA, Co-expression network, Developing brain transcriptome

## Abstract

**Background:**

Autism Spectrum Disorder (ASD) is the umbrella term for a group of neurodevelopmental disorders convergent on behavioral phenotypes. While many genes have been implicated in the disorder, the predominant focus of previous research has been on protein coding genes. This leaves a vast number of long non-coding RNAs (lncRNAs) not characterized for their role in the disorder although lncRNAs have been shown to play important roles in development and are highly represented in the brain. Studies have also shown lncRNAs to be differentially expressed in ASD affected brains. However, there has yet to be an enrichment analysis of the shared ontologies and pathways of known ASD genes and lncRNAs in normal brain development.

**Results:**

In this study, we performed co-expression network analysis on the developing brain transcriptome to identify potential lncRNAs associated with ASD and possible annotations for functional role of lncRNAs in brain development. We found co-enrichment of lncRNA genes and ASD risk genes in two distinct groups of modules showing elevated prenatal and postnatal expression patterns, respectively. Further enrichment analysis of the module groups indicated that the early expression modules were comprised mainly of transcriptional regulators while the later expression modules were associated with synapse formation. Finally, lncRNAs were prioritized for their connectivity with the known ASD risk genes through analysis of an adjacency matrix. Collectively, the results imply early developmental repression of synaptic genes through lncRNAs and ASD transcriptional regulators.

**Conclusion:**

Here we demonstrate the utility of mining the publically available brain gene expression data to further functionally annotate the role of lncRNAs in ASD. Our analysis indicates that lncRNAs potentially have a key role in ASD due to their convergence on shared pathways, and we identify lncRNAs of interest that may lead to further avenues of study.

**Electronic supplementary material:**

The online version of this article (10.1186/s12918-018-0639-x) contains supplementary material, which is available to authorized users.

## Background

Long non-coding RNAs (lncRNAs) are defined as transcripts greater than 200 nucleotides in length, which do not code for proteins. They serve a wide range of functions including, but not limited to, scaffolding for protein complexes, transcriptional regulation, and translational regulation [1–3]. Currently, the GENCODE consortium lists 15,941 lncRNA genes [4]. LncRNAs are potentially key regulators of brain development. Expression of lncRNAs has been shown to have increased temporospatial specificity in comparison to protein-coding genes, and lncRNAs are expressed in the brain at relatively high levels [5, 6]. Nescula et al. [7] found that lncRNA genes of earlier evolutionary origin have been shown to contain homeobox transcription factor binding sites in their promoter regions at a frequency greater than two times that of protein coding genes. This indicates the potential role of lncRNAs in development. This group also found that younger lncRNAs, in terms of phylogenic split from a common ancestor, show lower interspecies conservation and a number of lncRNA families unique to primates offer potential insight into higher cognitive functions.

Autism spectrum disorders (ASD) are a heterogeneous group of neurodevelopmental disorders with a complex genetic etiology. The diagnosis is determined by significant deficit in reciprocal social interactions, impaired communication, and restricted, repetitive behaviors, and most documented cases are clinically diagnosed by the age of three [8]. There is strong evidence to support a genetic causation model, including 88% pairwise concordance amongst monozygotic twins and 18.7% risk of ASD for siblings of affected individuals [9–11]. As with most complex genetic disorders, ASD could result from the accumulation of low risk common variants, high risk rare variants, or both. Approaches for ASD genetic studies have included copy number variation (CNV) studies, genome-wide association studies (GWAS) and rare de novo variant (RDNV) exome studies [11]. Ziats and Rennert [12] found 222 differentially expressed lncRNAs in ASD. ASD risk genes are convergent on synaptic gene translation, transcription and chromatin remodeling [11, 13], and these three processes can be controlled by lncRNAs [14].

This study used co-expression network analysis to identify lncRNAs potentially associated with ASD and provide possible functional annotations of lncRNAs for brain development. Since anatomical differences between ASD and control brain samples have been shown in several different structures, it is therefore beneficial in this study to examine all of the structures during the developmental period to place lncRNAs in a functional context within the developing brain [15]. The BrainSpan dataset offers a unique opportunity for identification of high-priority potential ASD associated lncRNAs owing to the comprehensive array of brain structures and developmental time points [16]. We have compiled a comprehensive list of ASD risk genes from several sources to measure co-expression with lncRNA genes annotated in the GENCODE dataset [4]. Co-expression network analysis was performed on a curated set of genes from the BrainSpan dataset to cluster the genes into modules. Expression patterns and co-enrichment with lncRNA genes and ASD risk genes were used to identify modules of interest. Enrichment analysis and network topology analysis were carried out to associate biologically significant functions with the modules. Finally, to identify lncRNA genes of interest, lncRNAs were prioritized based upon their association with the known ASD risk genes within the network.

## Methods

### Datasets

The BrainSpan dataset is a developmental transcriptome for the human brain [16]. It is an RNAseq dataset in units of reads per kilobase per million (RPKM), mapped to genes as annotated by the GENCODE consortium version 10. It consists of 524 samples covering a developmental time span of 8 weeks post conception to 40 years of age and 26 brain structures. Genes which did not show a minimum expression of 1 RPKM for at least one of the 524 samples and genes not present in the latest build of the GENCODE consortium (version 24) were removed from the dataset [4]. Expression values were then *log*_2_(*RPKM* + 1) transformed. Next, the sum of pairwise covariance was calculated for each gene. Using the KMeans class from the Scikit-learn Python library [17], clustering with the total clusters set to 2 was performed on the sum of covariance values to filter out low information genes [18, 19]. Then ASD risk genes and lncRNA genes within the dataset were identified (Additional file 1). ASD risk genes were compiled from three different sources. We selected 290 genes from the Gene Scoring Module from the Simons Foundation Autism Research Initiative (SFRARI) on the criteria of a score of 1–4 with 1 being high confidence and 4 being minimal evidence [20]. An additional 170 genes were from the core set of the Autism Knowledge Base from the Center for Bioinformatics in Peking University [21]. The third source from which we selected 107 genes was from an exome sequencing study for de novo loss-of-function mutations in ASD cases [22]. Redundancy among the three datasets were removed resulting in an ASD risk gene set consisting of 433 genes. Genes of the lncRNA biotypes were indicated in the GENCODE build.

### Co-expression network analysis

Genes were clustered into modules using the weighted gene co-expression network analysis (WGCNA) package in R [23]. The package first generates a topological overlap matrix using neighbourhood analysis and the weighted pairwise correlation between genes |*corr*(*x*_*i*_, *x*_*j*_)|^*P*^ where P is a soft threshold for network scalability. In this study, we found that a scale free topology was reached with a soft threshold of 7. Then a dissimilarity dendrogram from the topological overlap matrix is created, and the genes are grouped using a dynamic tree-cutting algorithm. The network in this study was an unsigned bi-weight network with a minimum module size of 30 and a merge cut-off height of 0.2. The heatmap of the expression patterns for the modules was generated using the gplots package in R [24]. The expression patterns themselves are the eigengene (first principal component) for the respective modules. Enrichment of lncRNA and ASD risk genes within the modules was calculated by applying Fisher’s exact test to gene type frequency within the module compared to gene type frequency for the entire dataset. The *P*-value was adjusted to a false discovery rate (FDR) to account for multiple testing using the p.adjust function in the stats package in R [25]. For better visualization of enrichment, significance values were −*log*_10_(*FDR*) transformed.

### Enrichment analysis

Functional term enrichment for each module was implemented through the use of the Database for Annotation, Visualization and Integrated Discovery (DAVID) [26]. This software receives a gene list and applies the EASE algorithm, which is a variation of the Fisher’s exact test, using gene annotations present in the database and a designated background. In this study, enrichment was measured against a human genome background, and genes which could not be mapped were not considered in the enrichment calculations. The FDR values generated from DAVID were transformed as mentioned previously. Enrichment for significantly expressing genes within brain structures was calculated using the same methodology used to determine ASD and lncRNA gene enrichment in the previous section. Frequencies were grouped by developmental time periods and gene types. The three developmental time periods used were prenatal (8pcw-37pcw), childhood (4mos-15 yrs), and adulthood (18 yrs–40 yrs) (pcw = post conception weeks; mos = months; yrs. = years). The gene types were lncRNA genes, ASD risk genes, and all genes within the module. The values were determined by the number of genes with expression greater than or equal to 1 RPKM for a given sample divided by the total possible number of genes compared to the appropriate background.

### Network visualization and analysis

To visualize a co-expression network, we first sought to determine significant interactions between genes. We constructed an adjacency matrix for the entire dataset using the absolute Pearson product moment correlation to a power of 7 as a measure of connectivity between genes. We then selected the top 5% of the correlations which became the edges in our network while the genes became the nodes. The network was then sub-divided based upon module assignments to determine changes in topology specifically in regard to lncRNA gene and ASD risk gene interactions. Representative modules were visualized using the Cytoscape software [27]. To prioritize lncRNA genes within our dataset for ASD association, we adapted a methodology used by Oliver et al. [28], which used connectivity as a means of prioritization. Here we sum the pairwise connectivity from the adjacency matrix between the target lncRNA gene and all the known ASD risk genes in the dataset. The connectivity score is then normalized using $\frac{\left({x}_i-\mathit{\min}(x)\right)}{\left(\mathit{\max}(x)-\mathit{\min}(x)\right)}$ for the range of all lncRNA genes analysed.

## Results

### Co-expression network analysis within the developing brain shows high co-enrichment of lncRNA genes and ASD risk genes in elevated pre- and postnatal expression modules

**Fig. 1:**
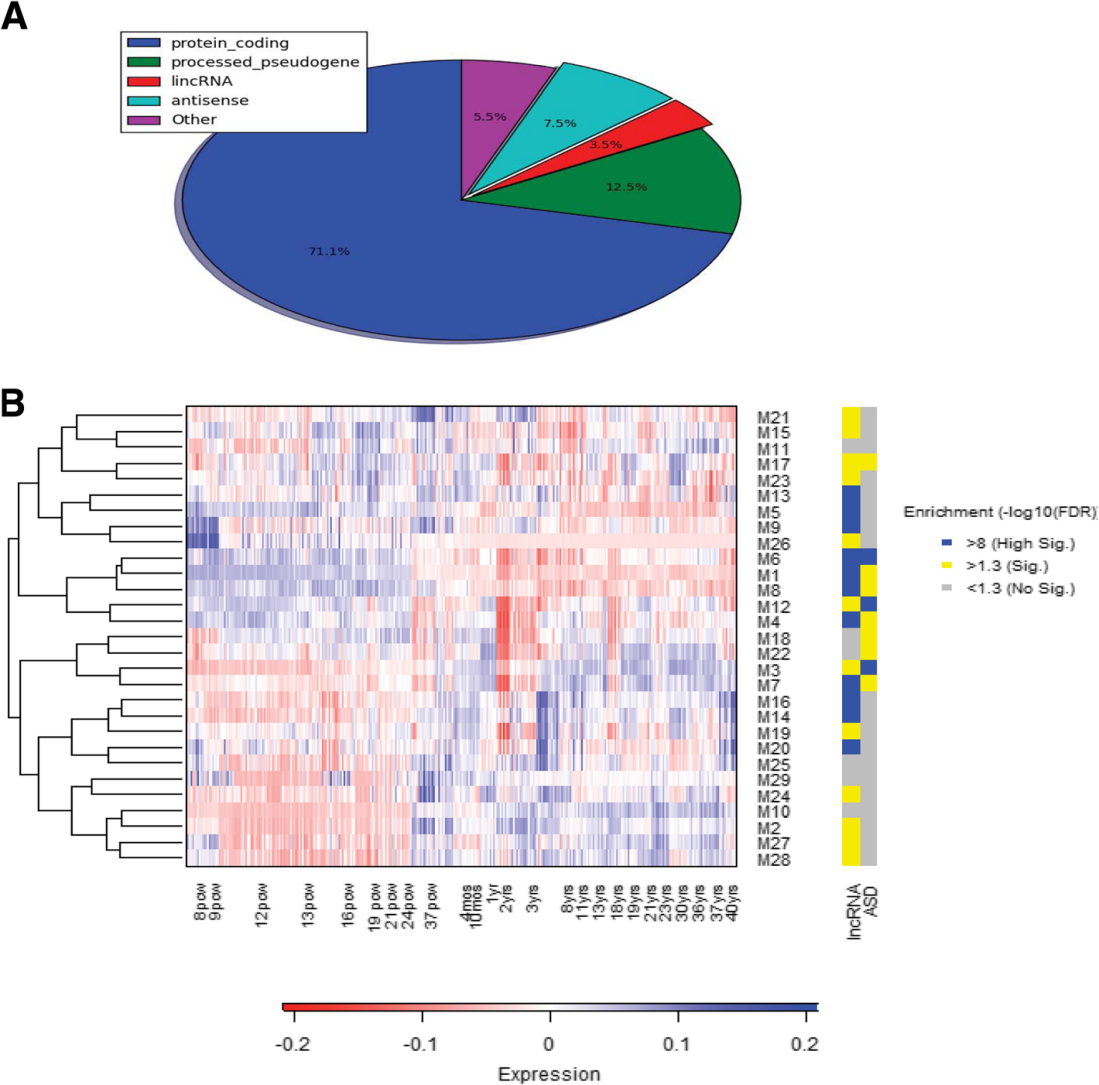
Gene co-expression analysis using the BrainSpan dataset. **a** Pie chart of the distribution of genes by biotype for the curated gene set. **b** Heatmap of module eigengenes from co-expression analysis for all samples chronologically. The row labels correspond to the module (M1 = Module 1) and the column labels indicate the time point ranges (pcw = post conception weeks, mos = months, and yrs. = years). To the right of the heatmap is a color sidebar mapping the enrichment of the modules for lncRNA genes and ASD risk genes respectively. The legend indicates the level of significance with the threshold set at 1.3 or FDR = 0.05 (sig = significance)

The BrainSpan dataset offers an opportunity to analyse in depth the gene expression patterns of the developing human brain [16]. However, the dataset required further curation for efficient co-expression analysis to prevent noise from low-expression or low-variance genes. We first removed the genes which did not show sufficient expression (< 1 RPKM) in all the samples, and then selected for genes which showed high pairwise covariance with other genes within the dataset. The 20,456 genes which remained after the curation presented an interesting distribution of gene biotypes (Fig. 1a). While protein-coding genes account for only 40.4% of the genes within the original dataset, after curation they account for 71.1% while antisense lncRNA genes and long intervening RNA (lincRNA) genes originally comprised 19.1% of the dataset were reduced down to 11%.

After dataset curation, weighted gene co-expression network analysis (WGCNA) was performed [23]. We found 29 modules. We mapped the expression pattern of the eigengene (first principal component) for each module onto a hierarchically clustered heatmap to better visualize shared expression patterns. Coenrichment for lncRNA genes and ASD risk genes were also mapped to the modules (Fig. 1b). We found two distinct clades from the module clustering, which show coenrichment for lncRNA genes and ASD risk genes. One clade comprised of modules M1, M4, M6, M8 and M12, which account for 9355 genes within the dataset, shows elevated expression in prenatal samples and lower expression in postnatal samples. These sets are further referred to in this paper as early expression modules. Intriguingly, the other clade comprised of modules 3 and 7 shows an inverse pattern in that prenatal expression is low and postnatal expression is elevated. These sets are referred to as late expression modules. Only 5 of the 29 modules did not show significant enrichment (FDR < 0.5) for lncRNA genes while 10 of the modules showed significant enrichment for ASD risk genes with module 6 being alone in showing high enrichment for both gene types.

### Enrichment analysis of two module groups shows term enrichment for transcriptional regulation and synapse formation respectively and complementary structure enrichment for sensory cortical regions

**Fig. 2:**
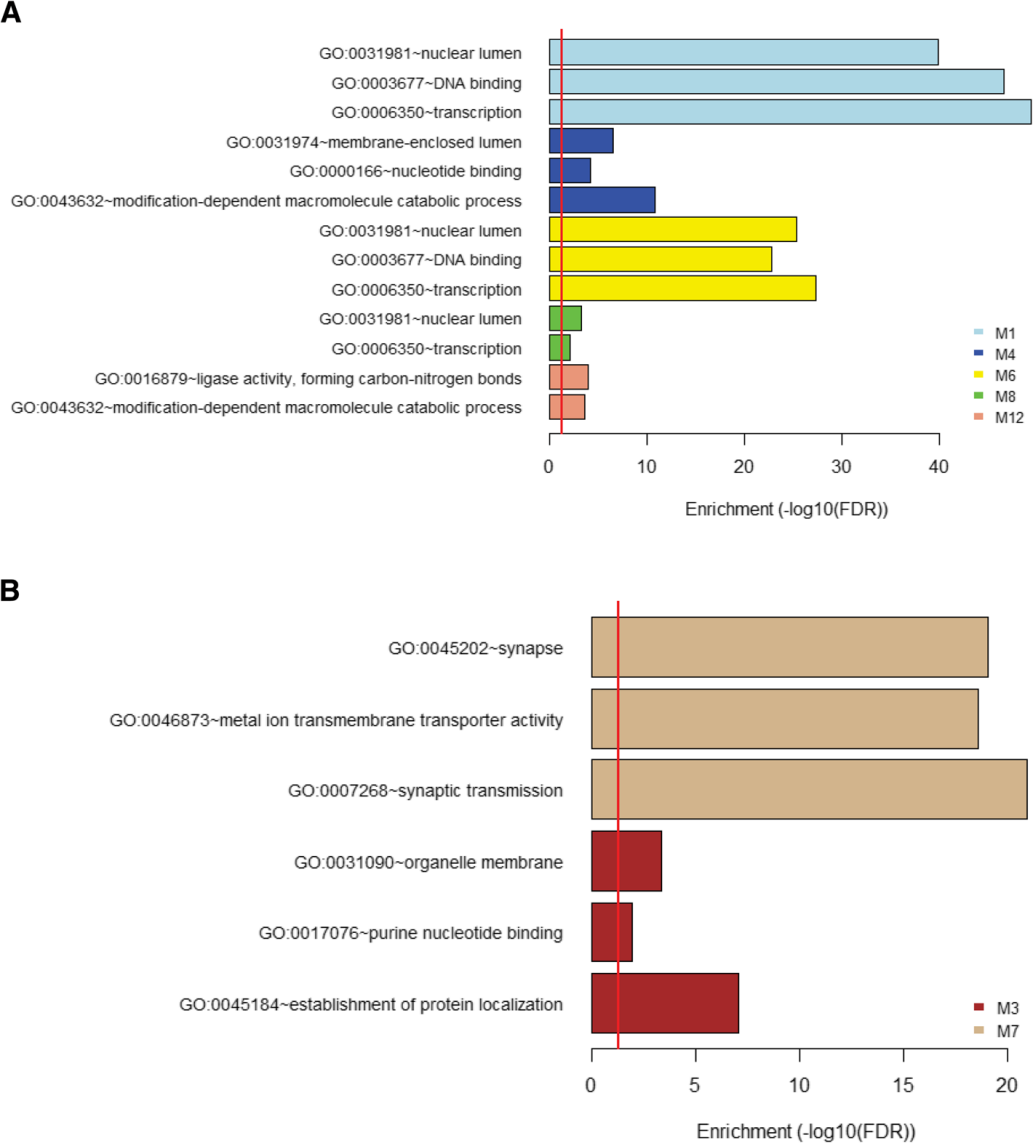
Term enrichment analysis of early and late expression module groups. **a** Term enrichment color coded by module for early expression modules. Term categories are from the top to bottom: cellular component, molecular function, and biological function for each module in the group. The red vertical line indicates the significance cutoff (FDR = 0.05). Modules 8 and 12 did not have significant terms for molecular function and cellular compartment respectively. **b** Term enrichment for late expression modules

To further characterize our module groups of interest, we performed term enrichment analysis. The Database for Annotation, Visualization and Integrated Discovery (DAVID) term enrichment analysis assigns Gene Ontology (GO) terms based upon their enrichment within the gene set [26]. It should be noted that the gene sets are comprised of all of the genes within a module and not limited to ASD and lncRNA genes. While there are several categories for terms, we chose biological process, molecular function, and cellular component functional annotation terms to characterize our module groups. These categories offer the most relevant information for lncRNAs whereas the other categories are more relevant to protein coding genes or partially redundant to the given categories. For each module in either the early expression or late expression group, the most significant terms for each category are shown in Fig. 2a and b. The early expression modules (M1, M4, M6, M8, and M12) show overlap in biological processes for the broad terms of transcription and modification-dependent macromolecule catabolic process, which corresponds to the breakdown of large macromolecules. There is also overlap in localization to the nuclear lumen, and the molecular function of DNA-binding as well as general nucleotide binding. Collectively this implies that the early expression modules are enriched for transcriptional regulators as well as partially involved in the breakdown of nucleotides. However, the late expression modules (M3 and M7) are enriched for a different aspect of brain development. While module 7 has enrichment for relatively ambiguous terms associated with protein transport, module 3 shows enrichment for genes involved in synaptic transmission and localized to the synapse.

**Fig. 3:**
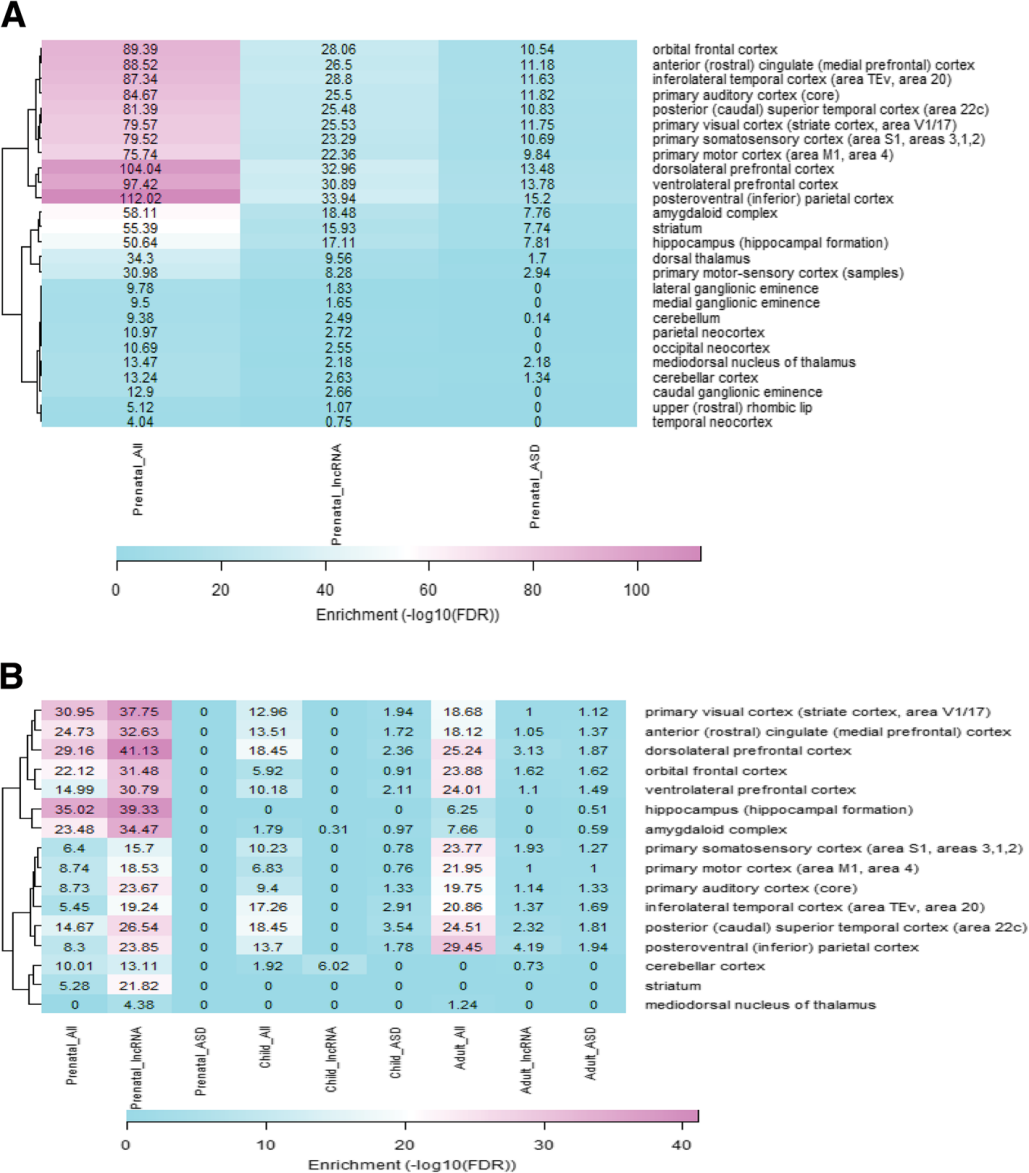
Gene expression enrichment analysis of early and late expression module groups. **a** Heatmap of brain structure enrichment for early expression modules. Row labels indicate the brain structure and the columns indicate the developmental period and the gene type with all corresponding to all of the genes within the module. The enrichment values are shown in each cell, and rows are clustered based on their enrichment values. **b** Heatmap of brain structure enrichment for late expression modules

Grouping together the samples based on brain structures and developmental periods (prenatal, childhood, and adulthood), we analysed the enrichment of structures for expressed genes collectively, lncRNA genes, and ASD risk genes for the two module groups (Fig. 3a and b). Some structures had samples for the prenatal period but did not have samples for childhood and adulthood. Therefore, for the early expression modules, we analysed all the structures for just the prenatal period as the later developmental periods (childhood and adulthood) showed little to no enrichment for structure-specific expression. For the late expression modules, we analysed only structures present in all three developmental periods.

Enrichment of expressed genes in the early expression modules was significant for all of the structures. Interestingly, expressed lncRNA genes and ASD risk genes show similar patterns of enrichment in the different brain structures for the early expression modules. Not surprisingly, expressed lncRNA genes and ASD risk genes are highly enriched for the sensory cortical regions, striatum, and amygdaloid complex. These three structures have been implicated in ASD [29–31].

Enrichment of expressed genes in the brain structures for the late expression modules shows distinct patterns based on gene type. With the exception of the mediodorsal nucleus of thalmus, all of the structures were significantly enriched (FDR < 0.05) for expressed genes during the prenatal period. Thirteen of the structures were significantly enriched for expressed genes during childhood, and 13 structures were significantly enriched for expressed genes during adulthood. The hippocampus became significantly enriched during the transition from childhood to adulthood, and the cerebellar cortex lost significant enrichment in the same transition period. Enrichment values for expressed lncRNA genes and ASD risk genes within structures do not show the same similarities as was observed for the early expression modules. Expressed lncRNA genes are significantly enriched in the prenatal period for every structure, only significantly enriched for the cerebellar cortex in the childhood developmental period, and significantly enriched for six structures in the adult developmental period. Expressed ASD risk genes show no enrichment for any structure in the prenatal period, significant enrichment in eight structures during the childhood developmental period, and significant enrichment in eight structures during the adult developmental period. Intriguingly, there is no significant enrichment for expressed ASD risk genes in the striatum, which has been implicated in the physiopathology of ASD [29].

### Visualization of the topology of module networks demonstrates high connectivity between lncRNA genes and ASD risk genes in early expression modules

**Fig. 4:**
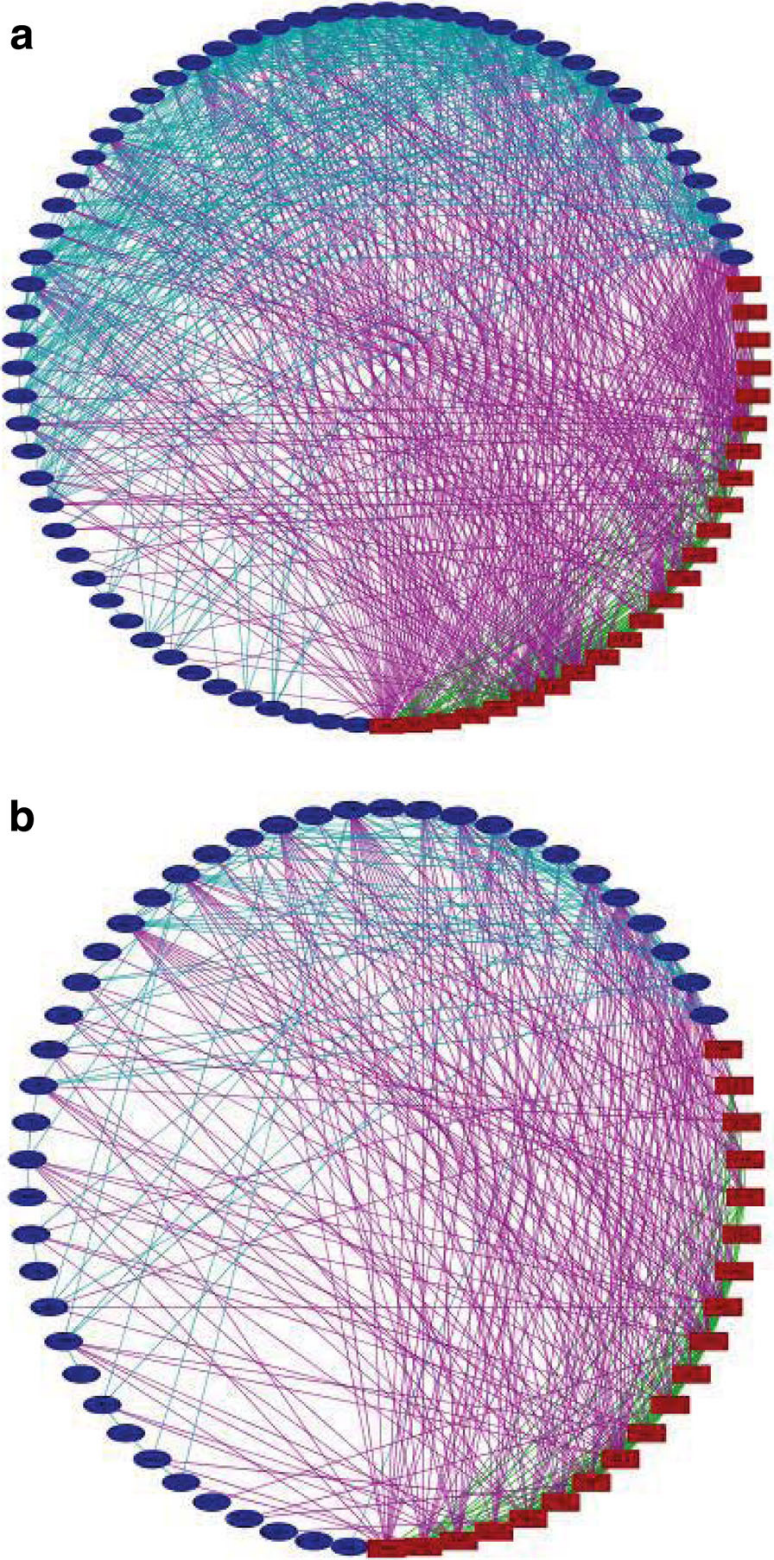
Co-expression network topology for modules of interest. **a** Network topology for lncRNA genes and ASD risk genes in module 12. ASD genes are red rectangles, and lncRNA genes are blue ellipses. Interactions are color coded as follows: ASD to ASD = Green, ASD to lncRNA = Purple, lncRNA to lncRNA = turquoise. Modules are in an attribute based circular layout with the attribute being gene type. **b** Network topology for lncRNA genes and ASD risk genes in module 7

While term and structure enrichment can give general information about the biological roles associated with modules and provide potential annotation for uncharacterized lncRNA genes, it does not indicate the interactivity between ASD risk genes and lncRNA genes. The enrichment analysis did demonstrate the possibility that lncRNAs and ASD risk genes may be more closely associated in the early expression modules than in the later ones. This is confirmed by network analysis. To form the network, we used an adjacency matrix to establish pairwise correlation for all of the genes. We then selected for the most significant (highly correlated) interactions for the network. For each of the modules of interest, we observed the significant connections between lncRNA genes and ASD risk genes and found that for the early expression modules there was greater connectivity between the two gene types. Figure 4a and b show the networks for a representative early expression module (M12) and late expression module (M7) respectively. One module from each group was chosen to demonstrate the contrast in topologies between them. Module 12 shows dense connectivity for all of the nodes but does have a greater number of interactions between ASD risk genes and lncRNA genes than between lncRNA genes and ASD risk genes respectively. Notably, there is a high degree of interaction between lncRNA genes. However, module 7 shows a less dense network even though the number of genes present is comparable to that of module 12. Interactions between ASD risk genes and lncRNA genes are greater than other interactions in the network with few interactions between lncRNA genes.

### Prioritization of candidate lncRNA genes using connectivity with known ASD risk genes implicates biologically relevant targets

To identify high-priority targets for further study, we prioritized the lncRNA genes in our dataset based on their connectivity with known ASD risk genes. For each lncRNA gene, the pairwise correlation from our adjacency matrix was summed for all ASD risk genes. Genes were ranked relative to their sums of connectivity with higher values associated with greater potential association with ASD. The complete list of lncRNAs with their module assignment and normalized values for ASD gene connectivity and adjusted intramodular connectivity are provide in Additional file 1. Adjusted intramodular connectivity is the sum of the pairwise connectivity of a gene for all other genes within the module with the sum of pairwise connectivity for the gene for all genes not in the module subtracted from it. The normalized value is based upon the range for all the genes in the dataset and calculated using the same method as for the normalized value for ASD gene connectivity. Table 1 shows highly prioritized lncRNA genes for tentative links to ASD. The gene prioritized the highest is RP11-281C10.5, an antisense lncRNA to CEP170, which is a component of the centromere and critical to cell division [32]. KDM4A-AS1 is antisense to KDM4A, a lysine de-methylase, which has been shown to increase copy number gains in CNVs associated with ASD [33]. LINC-PINT is a lincRNA, which is activated by P53 and like the well-characterized lncRNA, HOTAIR, has been shown to associate with polycomb recessive complex 2 [34]. TUG1 is one of the few highly prioritized genes not grouped to module 1, and it has low intramodular connectivity. It has no direct link to ASD but has been implicated in neurodegenerative disorders [35]. The role of lncRNAs in ASD is still being elucidated, so it is not surprising that many of the genes have no direct link to ASD. The list itself acts as a putative implication of the highly prioritized lncRNAs for their role in ASD.

**Table 1:** List of selected biologically significant and highly prioritized lncRNA genes for ASD association

Name	Biotype	ASD Connectivity (Normalized)	Module	Intramodular Connectivity (Normalized)
RP11-261C10.5	Antisense	1	1	0.8482
KDM4A-AS1	Antisense	0.9015	1	0.8479
LINC-PINT	Antisense	0.7091	1	0.7510
TUG1	Antisense	0.6992	6	0.3157

## Discussion

With the relatively recent expansion of autism to include Asperger’s Syndrome, Rett Syndrome, uncharacterized pervasive developmental disorders, and Autistic Disorder under the common banner of Autism Spectrum Disorders (ASD), the complexity of finding its causality increases [8]. While there have been significant advances in clinical diagnostic tools, the number of ASD-affected individuals has increased at a rate greater than what is estimated to be due to improved diagnostics with the CDC reporting a 10-fold increase over a 20-year period [36]. There are competing theories on the underlying cause of the disorder, which are not mutually exclusive [11]. However, the leap from genetic abnormalities to phenotypic causation has been difficult due to a multitude of factors, including the difficulty of studying the brain physiology of affected individuals and the complexity of genetic interactions associated with the disorder. In this study, we utilized the most comprehensive expression dataset currently available for the developing human brain to further elucidate the complex interactions in an effort to show the role of lncRNAs in brain development and ASD.

LncRNAs have been shown to be in evolutionarily conserved gene families unique to primates and even further to humans alone [7], yet their functional roles in brain development warrant further definition. This study indicates a critical role for lncRNAs in transcriptional regulation and synaptic formation in the brain during development. In this study, we have broadly characterized the role of lncRNAs in brain development and ASD. Clustering our curated gene list, we found that lncRNAs were enriched nearly ubiquitously across our modules but only co-enriched with ASD risk genes in two distinct module groups showing high prenatal and high postnatal expression respectively. This distinction in expression at that particular developmental point is interesting as it has been previously implicated as a critical time for ASD development [13]. This data combined with term enrichment suggesting transcriptional regulation and the network topologies showing higher numbers of significant interactions between lncRNA genes and lncRNA genes strongly suggest that lncRNAs within the group of early expression modules regulate brain development through repression of genes controlling synapse formation possibly in the late expression modules.

ASD is a neurodevelopmental disorder, and in identifying potentially important lncRNA regulators of brain development we have also begun to identify putative high priority targets for potential therapeutics and diagnostics. Due to their tight regulatory control [5], lncRNAs are excellent biomarkers. One of the most notable examples was in 1995 when the lncRNA PCA3 was discovered and has since become a diagnostic for pancreatic cancer [37]. It was recently found that 90% of disease associated SNPs from genome-wide association studies were found outside of protein coding regions [14], which indicates non-coding genes and regulatory regions within the genome could have a major role in disease. These regions may also provide insight into the etiology of complex disorders such as ASD.

We have previously applied the approach of co-expression network analysis to define high priority disease-associated lncRNA genes based upon normal tissue expression patterns when we published work showing strong associations between cancer genes and lncRNAs [6]. Our approach allows for disease associations to be implied based solely on expression patterns. It is our hope that this study will highlight lncRNA genes that can act as diagnostic markers to the disorder as well as genes that can further elucidate the etiology of ASD. We also hope that this study further demonstrates the utility of co-expression network analysis on non-disease samples to implicate lncRNAs in disorders.

## Conclusions

In this study, we performed gene co-expression network analysis to identify candidate lncRNAs associated with ASD. Co-enrichment of lncRNAs and ASD risk genes was found in two distinct groups of modules showing different expression patterns and enriched functional terms. We prioritized the candidate lncRNAs based on their connectivity with the known ASD risk genes. The results suggest that lncRNAs may have a key role in ASD, and thus the prioritized list of candidate lncRNAs can be useful for further experimental studies to understand the etiology of ASD.

## Additional file

Additional file 1: List of lncRNAs with their module assignment and association with ASD risk genes in the co-expression network. (CSV 261 kb)

## Abbreviations

ASD: autism spectrum disorder
DAVID: database for annotation, visualization and integrated discovery
FDR: false discovery rate
lncRNA: long non-coding RNA
RPKM: reads per kilobase of transcript per million mapped reads
WGCNA: weighted gene co-expression network analysis
